# Adaptation of A-to-I RNA editing in bacteria, fungi, and animals

**DOI:** 10.3389/fmicb.2023.1204080

**Published:** 2023-05-24

**Authors:** Yuange Duan, Hu Li, Wanzhi Cai

**Affiliations:** Department of Entomology and MOA Key Lab of Pest Monitoring and Green Management, College of Plant Protection, China Agricultural University, Beijing, China

**Keywords:** A-to-I RNA editing, bacteria, evolution, adaptation, proteomic diversification

## A-to-I RNA editing is prevalent in all domains of lives

**Figure 1:**
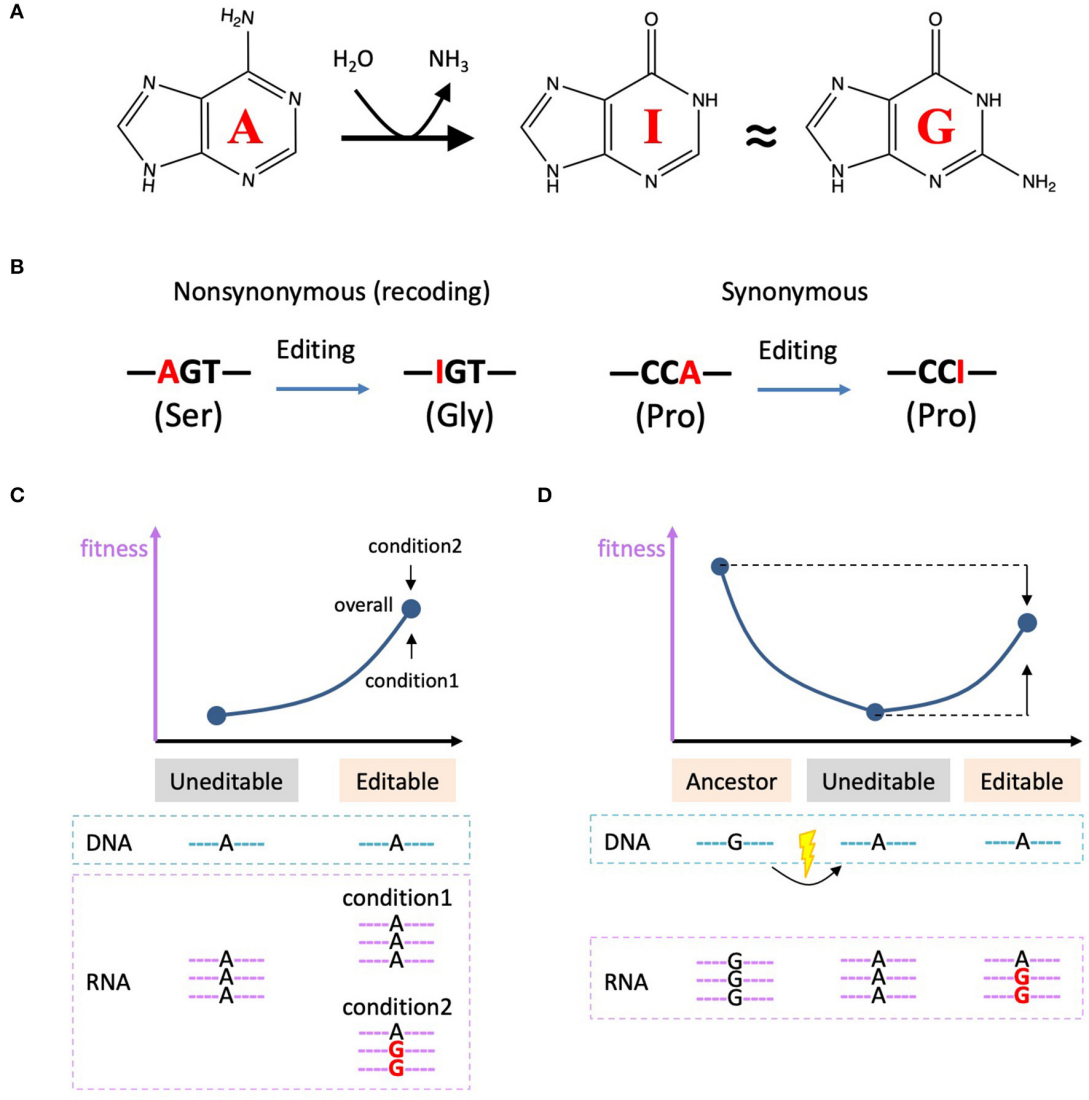
A-to-I RNA editing and the current theories on adaptive non-synonymous editing. **(A)** Chemical structures of adenosine, inosine, and guanosine and the process of A-to-I RNA editing. **(B)** A-to-I RNA editing in CDS is able to recode the genetic information. **(C)** Diversifying hypothesis stresses the advantage of the flexibility of RNA editing. We denote I as G in the schematic diagrams. **(D)** Restorative hypothesis states that the edited allele is no fitter than the ancestral genomic G-allele.

Adenosine-to-inosine (A-to-I) RNA editing is a prevalent type of RNA modification in all domains of lives ranging from bacteria (Liao et al., 2023), fungi (Liu et al., 2017), to metazoans (Duan et al., 2018; Zhang et al., 2023). Inosine is believed to be recognized as guanosine (G) (Figure 1A) and therefore A-to-I RNA editing is able to change genetic information at RNA level. In coding sequence (CDS), non-synonymous editing sites will cause recoding events that largely diversify the proteome (Eisenberg and Levanon, 2018) (Figure 1B). The adaptation of A-to-I RNA editing in bacteria, fungi, and animals has been systematically summarized by a recent paper (Liao et al., 2023). In this article, we will follow this topic and make an in-depth but concise discussion on the evolutionary significance, biological consequence, and adaptive signals of A-to-I RNA editing.

## A brief introduction of the RNA editing enzymes

Despite the deep conservation of the RNA editing mechanism in the tree of life, the enzymes responsible for A-to-I editing have remarkably evolved in different major branches. In metazoans, A-to-I mRNA editing is catalyzed by the ADAR (adenosine deaminase acting on RNA) protein family (Savva et al., 2012; Duan et al., 2022). In fungi, the editing enzyme is ADAT2/3 (adenosine deaminases acting on tRNA) rather than ADAR (Liu et al., 2016, 2017). In bacteria, TadA (tRNA-specific adenosine deaminase) or its isozyme is responsible for exerting A-to-I RNA editing (Bar-Yaacov et al., 2017; Nie et al., 2021). Moreover, the *cis* elements preferred by the editing enzymes are slightly different between different clades (Nishikura, 2010; Bian et al., 2019). Nevertheless, extensive recoding by A-to-I editing has been observed in the CDSs of a wide variety of species (Alon et al., 2015; Liscovitch-Brauer et al., 2017; Liu et al., 2017; Duan et al., 2021), dramatically diversifying the proteomes in a temporal-spatial manner.

## Two complementary hypotheses on adaptive non-synonymous A-to-I RNA editing

Despite the putative advantage of proteomic diversifying role of RNA editing, evolutionary biologists believe that one should be very cautious when declaring the “adaptation” of a biological process. A common misinterpretation on “adaptation” is “automatically regarding a biological consequence to be adaptive without clarifying the selective advantage, which is, how this mechanism increases the fitness of an organism?” (Graur et al., 2013). Therefore, to prove the adaptiveness of recoding events caused by non-synonymous RNA editing, we should find out exactly why the editable status is more advantageous than the uneditable status, and how RNA editing increases the fitness of an organism. The recent (Liao et al., 2023) paper has summarized two complementary theories on adaptive RNA editing.

The most classic theory is the “diversifying hypothesis” which states that RNA editing confers its advantage by diversifying the proteome in a flexible manner (Gommans et al., 2009; Duan et al., 2017) (Figure 1C). We denote I as G for simplicity. For the uneditable sites, the DNA sequence is adenosine and the corresponding RNA could not be edited. For the editable sites, the DNA is adenosine, but the RNA is able to selectively be adenosine under condition1 and could also be (partially) edited to guanosine under condition2. The editing level is flexibly adjusted according to which allele is favorable under a certain condition (Figure 1C). For example, under condition1, A-allele is better, so the organism increases the proportion of unedited mRNAs; under condition2, G-allele is better, so the organism increases the proportion of edited mRNAs (Figure 1C). Note that the G-allele is not always fitter than A-allele under the diversifying hypothesis, but the overall fitness is definitely higher for the editable status compared to the uneditable status (Figure 1C). The editability provides a flexible choice for the organism. Later we will mention that this hypothesis was recently experimentally verified in fungi (Xin et al., 2023).

In contrast, the “restorative hypothesis” proposes that A-to-I RNA editing aims to reverse the deleterious G-to-A mutation in DNA. It is assumed that G-allele is always fitter than A-allele. However, although G is better than A, the edited status in mRNA (with mixed G and A) is no fitter than the ancestral genomic G-allele (Figure 1D). This is why a non-adaptive conclusion was drawn by comparing the current state *vs*. the ancestral sate (Jiang and Zhang, 2019). Nevertheless, if one strictly refers to the RNA editing process itself (edited allele *vs*. unedited allele) rather than making a crosstalk between current RNA *vs*. ancestral DNA, then it is apparent that the editing mechanism still increases the fitness of the uneditable allele (Figure 1D). Under this scenario, this action/process of restorative RNA editing is adaptive (Duan et al., 2023).

## Genome-wide evidence for adaptation of non-synonymous A-to-I RNA editing in bacteria

Given these established theories on adaptive RNA editing, one should provide concrete evidence to support these hypotheses. Liao et al. has well summarized a few cases of functional A-to-I recoding in bacteria (Liao et al., 2023). For example, Bar-Yaacov et al. 2017 found 12 non-synonymous editing sites in *E. coli* that all change Tyr (TAC) to Cys (TGC), among which the recoding site in *hokB* gene affects the toxicity of the protein product and could improve the adaptation of bacteria population under antibiotic stress (Bar-Yaacov et al., 2017). In addition, the functional importance of two recoding sites, S128P and T408A, in bacterium *Xanthomonas oryzae* pv. *oryzicola* (*Xoc*) were shown (Nie et al., 2020, 2021; Liao et al., 2023).

**Figure 2:**
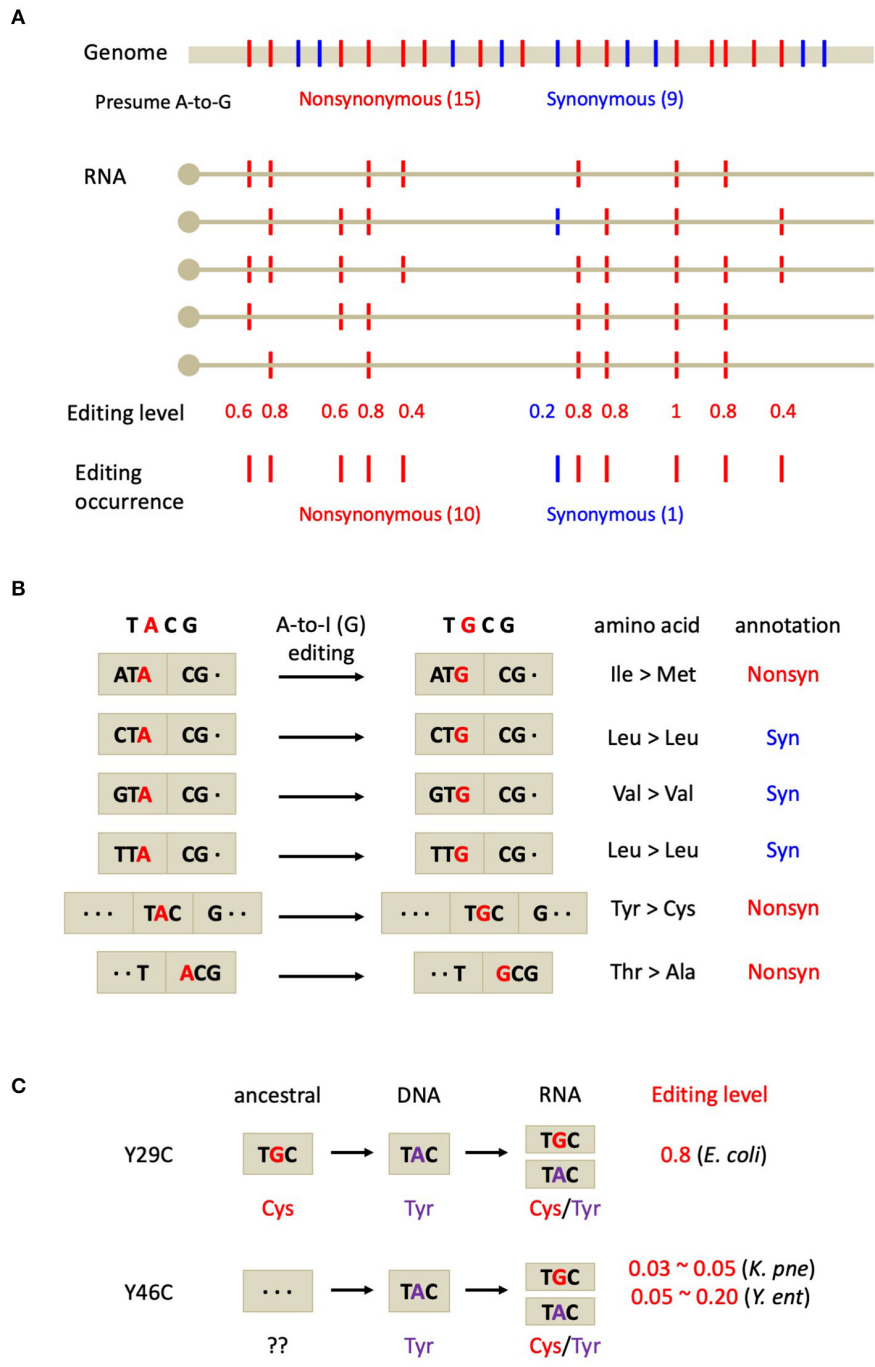
Testing the adaptive hypothesis on bacterial A-to-I RNA editing. **(A)** Commonly accepted criteria for adaptive RNA editing revealed from the genome-wide analysis. Non-synonymous editing shows higher occurrence and editing level than synonymous editing. **(B)** Expected fractions of non-synonymous and synonymous editing sites from the TACG motif in *E. coli*. **(C)** The evolutionary trajectory of the two Tyr-to-Cys recoding sites in bacteria. *K. pne, Klebsiella pneumoniae*; *Y. ent, Yersinia enterocolitica*.

However, an unsolved question is could we find solid evidence to prove the evolutionary adaptation of the global non-synonymous RNA editome instead of presenting case studies? Evolutionary biology particularly focuses on the global trend that has been shaped by long-term natural selection and therefore case studies do not add so much to the confidence of evolutionary theories. Notably, one should distinguish two terms “functional” and “adaptive”. For instance, although a few non-synonymous editing sites in mammals are functional (Sommer et al., 1991; Xu and Zhang, 2015), the overall non-synonymous sites are non-conserved and show no signals of positive selection (Xu and Zhang, 2014). Based on these knowledges, here is an intuitive inference on adaptation: if non-synonymous RNA editing is overall adaptive, then it should be positively selected and exhibit higher occurrence and editing levels than the neutral synonymous editing sites (Figure 2A). These commonly accepted criteria have been well applied to several animal species where non-synonymous editing is prevalent (Duan et al., 2022; Zhang et al., 2023).

In bacteria, however, mRNA editing is very rare (Bar-Yaacov et al., 2017) and is likely to be the by-product from tRNA editing (Liao et al., 2023). This weakens the statistical power in judging the adaptive signal of non-synonymous editing. Nevertheless, the fact that all the 12 editing sites in *E. coli* mRNA belong to non-synonymous sites (Bar-Yaacov et al., 2017) generally reflects a signal of adaption on recoding events (at genome-wide level). A simple logic is, if the mRNA editing in *E. coli* just randomly comes from the off-target events of tRNA editing, then the synonymous sites should be edited as well, exhibiting a non-synonymous/synonymous ratio of roughly 2/1 (expected by chance) rather than 12/0 (observed by Bar-Yaacov et al.). Even taken the editing-prone TACG motif into account, roughly half of the A-to-G events in the TACG motifs (in CDS) should be synonymous mutations (Figure 2B). Here are the detailed statistics regarding an adenosine in a TACG context: frame1: NTA-to-NTG (3 synonymous & 1 non-synonymous); frame2: TAC-to-TGC (1 non-synonymous); frame3: *A*CG-to-GCG (1 non-synonymous) (Figure 2B). Indeed, the actual fraction of expected non-synonymous/synonymous sites should fully consider the codon usage in the *E. coli* genome. However, it is obvious that the observed non-synonymous/synonymous ratio of 12/0 should be much higher than the neutral expectation under any criteria, suggesting that the global non-synonymous editing sites in *E. coli* are likely to be evolutionarily adaptive.

## Does non-synonymous editing in bacteria belong to diversifying or restorative hypothesis?

Given that the A-to-I RNA editome in *E. coli* contains excessive non-synonymous editing and thus exhibit signal of adaptation, a more fundamental question is, what is the nature of adaptation in bacteria? What is the advantage of recoding? Does non-synonymous editing conform with diversifying hypothesis or restorative hypothesis?

The diversifying hypothesis stresses the flexibility (condition-specificity) of RNA editing (Gommans et al., 2009). Apart from the well-acknowledged cases of adaptive recoding in insects (Yu et al., 2016; Duan et al., 2017) and cephalopods (Garrett and Rosenthal, 2012; Liscovitch-Brauer et al., 2017) that show temporal-spatial regulation, strong evidence was recently found to support the adaptive proteomic diversification of RNA editing in fungi (Xin et al., 2023). The pre-edit allele is beneficial in the asexual stage of *Fusarium graminearum* while the post-edited allele is beneficial in the sexual stage. Overall, the editable status is more advantageous than uneditable status (agreeing with the model in Figure 1C). The relative fitness of two alleles is experimentally measured using mutant strains (Xin et al., 2023). This is the first case to prove the advantage of flexible RNA editing over the genomically encoded adenosines or guanosines.

On the other hand, the restorative hypothesis requires the ancestral sequence of editing sites to be the post-edited version and that the editing level should be high. Restorative RNA editing typically takes place in plants (Duan et al., 2023) and whether it exists in animals is still under debate (Jiang and Zhang, 2019; Shoshan et al., 2021).

Regarding the nature of adaptive editing in bacteria, the case study of Tyr-to-Cys (Y29C & Y46C) recoding sites in bacteria *hokB* genes (Bar-Yaacov et al., 2017) are not informative enough to distinguish between the two hypotheses. However, evidence could be found from their editing profile combined with the phylogeny. The *E. coli* Y29C site in *hokB* seems to restore the ancestral amino acid (Cys) but in the *E. coli* genome this site is already fixed as Tyr (Bar-Yaacov et al., 2017) (Figure 2C). Accordingly, the editing level of Y29C site is as high as ~80%, agreeing with the prediction made by the restorative hypothesis (Duan et al., 2023). In contrast, the evolutionary trajectory of another Y46C recoding site in two bacterial species (*Klebsiella pneumoniae* and *Yersinia enterocolitica*) is highly complicated (Bar-Yaacov et al., 2017). At position 46 of *hokB* gene in different bacteria, the two genomically encoded amino acids Tyr and Cys are totally mixed in the phylogeny, making it difficult to infer the ancestral state of the editing site (Figure 2C). Moreover, the editing level of Y46C recoding site in *Klebsiella pneumoniae* is roughly 3~5% (Bar-Yaacov et al., 2017). This low editing level hardly convinces us that this editing site is used for correcting a deleterious DNA mutation. Only editing 3% of the mRNAs could not rescue the majority (97%) of the deleterious allele. On the contrary, low editing level usually supports the diversifying hypothesis. Therefore, to distinguish between the diversifying *vs*. restorative hypotheses of bacterial RNA editing, it needs (1) more systematic identification of RNA editing sites, (2) higher resolution of editing levels, and (3) more accurate inference of the ancestral state of each editing site. Nevertheless, we do not exclude the possibility that in bacteria, some of the RNA editing sites play a diversifying role while some other editing sites restore the ancestral sequence.

## Other factors affecting the selection and evolution of RNA editing in bacteria

Given the ambiguity of the nature of adaptive RNA editing in bacteria, we try to figure out the potential factors shaping the function, natural selection, and evolution of bacterial RNA editing. Firstly, the strength of natural selection is associated with the ploidy of the genome. Haploid genomes lack the recombination mechanism and are less tolerant to the deleterious mutations compared to diploid and polyploid genomes. Bacteria are mostly haploid. Fungi are either haploid, diploid, or polyploid according to different stages. Animals are mostly diploid except Hymenoptera (bees and ants) that has haploid males. Therefore, compared to fungi and animals, bacterial genomes are subjected to stronger purifying selection. Although A-to-I RNA editing occurs in RNA rather than the genome, the editing efficiency (probability) is determined by the sequence context, which is affected by genomic mutations. The consequence is, the evolution of RNA editing is tightly connected to genome evolution (Liscovitch-Brauer et al., 2017; Duan et al., 2018). Constraint on genomic mutation will finally limit the emergence of novel RNA editing events. Under this scenario, it is not surprising to observe that mRNA editing is extremely rare in bacteria. The ambiguity in the adaptive signals of bacterial RNA editing became inevitable given the insufficient data size of RNA editing sites.

Next, as introduced, bacterial TadA is responsible for A-to-I editing on both tRNAs and mRNAs (Bar-Yaacov et al., 2017; Nie et al., 2021), while in animals, mRNA editing is specifically exerted by ADARs and tRNA is edited by ADAT (Bar-Yaacov et al., 2018; Duan et al., 2022). Interesting, the A_34_-to-I_34_ tRNA editing (in the first position of anticodon) is designed for achieving the wobble pairing between I-C, I-U, and I-A (anticodon-codon), enabling a tRNA to be potentially decoded by multiple synonymous codons (dos Reis et al., 2004). In fact, the “base-pairing potential” of I_34_ in tRNAs is wider than that of inosines in mRNAs. Interestingly, presume that if this base-pairing property of tRNA I_34_ is completely applied to mRNA editing, then inosines could be decoded as either G, A, or U, dramatically diversifying the amino acids at editing sites. Since tRNAs and mRNAs are edited by the same enzyme TadA in bacteria, we reserve the possibility that inosines in bacterial mRNAs could base-pair with more than cytidines. This scenario, where A-to-I editing simultaneously resembles A-to-G/U/A, would largely diversify the proteome beyond the traditional diversifying hypothesis.

## Summary

In summary, there are two complementary hypotheses (diversifying and restorative) to explain the nature of adaptive non-synonymous RNA editing. Through years of studies, the scenario of genome-wide adaptive editing is relatively clear in fungi, animals, and even plants. In bacteria, however, due to the limited number of discovered editing sites, the adaptive signals are elusive. By re-analyzing the A-to-I RNA editing sites in *E. coli*, we propose that the non-synonymous editing is likely to be positively selected. Nevertheless, more delicate investigation is needed to obtain a solid conclusion regarding the nature of adaptive RNA editing in bacteria.

## Author contributions

Conceptualization and supervision: WC and HL. Writing—original draft: YD. Writing—review and editing: YD, WC, and HL. All authors contributed to the article and approved the submitted version.
